# Inhibition of Tanshinone IIA, Salvianolic Acid A and Salvianolic Acid B on Areca Nut Extract-Induced Oral Submucous Fibrosis *in Vitro*

**DOI:** 10.3390/molecules20046794

**Published:** 2015-04-15

**Authors:** Jian-Ping Dai, Dan-Xia Zhu, Jiang-Tao Sheng, Xiao-Xuan Chen, Wei-Zhong Li, Ge-Fei Wang, Kang-Sheng Li, Yun Su

**Affiliations:** 1 Department of Microbiology and Immunology, Shantou University Medical College, Shantou 515041, China; E-Mails: scorpion37@126.com (D.-X.Z.); shengjt@126.com (J.-T.S.); chen.x.x.85@163.com (X.-X.C.); gfwangstdx@sina.cn (G.-F.W.); 2 Department of Veterinary Medicine, University of Maryland, College Park, MD 20742, USA; E-Mail: wzlistdxedu@sina.cn

**Keywords:** tanshinone IIA1, salvianolic acid A, salvianolic acid B, areca nut, oral submucous fibrosis

## Abstract

*Salvia miltiorrhiza* Bunge has been reported to possess excellent antifibrotic activity. In this study, we have investigated the effect and mechanism of tanshinone IIA1 (Tan-IIA1), salvianolic acid A (Sal-A) and salvianolic acid B (Sal-B), the important active compounds of *Salvia miltiorrhiza* Bunge, on areca nut extract (ANE)-induced oral submucous fibrosis (OSF) *in vitro*. Through human procollagen gene promoter luciferase reporter plasmid assay, hydroxyproline assay, gelatin zymography assay, qRT-PCR, ELISA and Western blot assay, the influence of these three compounds on ANE-stimulated cell viability, collagen accumulation, procollagen gene transcription, MMP-2/-9 activity, MMP-1/-13 and TIMP-1/-2 expression, cytokine secretion and the activation of PI3K/AKT, ERK/JNK/p38 MAPK and TGF-β/Smads pathways were detected. The results showed that Tan-IIA1, Sal-A and Sal-B could significantly inhibit the ANE-stimulated abnormal viability and collagen accumulation of mice oral mucosal fibroblasts (MOMFs), inhibit the transcription of procollagen gene *COL1A1* and *COL3A1*, increase MMP-2/-9 activity, decrease TIMP-1/-2 expression and inhibit the transcription and release of CTGF, TGF-β1, IL-6 and TNF-α; Tan-IIA1, Sal-A and Sal-B also inhibited the ANE-induced activation of AKT and ERK MAPK pathways in MOMFs and the activation of TGF-β/Smads pathway in HaCaT cells. In conclusion, Tan-IIA1, Sal-A and Sal-B possess excellent antifibrotic activity *in vitro* and can possibly be used to promote the rehabilitation of OSF patients.

## 1. Introduction

It has been reported that there are about 600 million betel quid chewers around the world [1], and epidemiologic studies have indicated that betel quid chewing is a major risk factor of oral submucous fibrosis (OSF), oral leukoplakia and oral squamous cell carcinoma [2]. OSF is a chronic inflammatory disease that can cause difficulty in chewing, swallowing, speaking and mouth opening. The important histopathological characteristic of OSF is the abnormal deposition of collagen in the oral submucosa [3]. Various findings indicate that the disease is a consequence of disturbances in the homeostatic equilibrium between synthesis and degradation of extracellular matrix (ECM), and OSF can be considered as a collagen-metabolic disorder disease [4,5,6]. OSF is closely associated with areca nut chewing [7]; areca nut extract (ANE) can induce the production of TGF-β, IL-6 and prostaglandin E2, upregulate the expression of COX-2 and heat shock protein 27 and activate PI3K/Akt, PKC-α, ERK/JNK/p38 MAPK, NF-κB and TGF-β/Smads signaling pathways, some of which contribute to the malignant transformation of OSF [8,9,10,11,12,13].

In our study, we have developed a chewable tablet to treat OSF, in which one of the major medicines is *Salvia miltiorrhiza* Bunge [5,14]. *Salvia miltiorrhiza* Bunge, a traditional Chinese medicine, has antifibrotic, antioxidative, anti-inflammatory and antiapoptotic properties; it can ameliorate hepatic, pulmonary and cardiac fibrosis by suppressing the expression of type I/III collagen, α-SMA, CTGF, TGF-β, TNF-α, IL-1α and caspase-3, upregulating the expression of MMP-2/-9, decreasing MDA production and increasing SOD activity [15,16]. *Salvia miltiorrhiza* Bunge contains ethanol-soluble and water-soluble active ingredients. An important ethanol-soluble compound is tanshinone IIA1 (Tan-IIA1); it can inhibit hepatic, pulmonary and cardiac fibrosis via inhibiting the activation of PI3K/AKT, ERK/JNK/p38 and NF-κB pathways [17,18]. The important water-soluble active compounds include salvianolic acid A (Sal-A) and salvianolic acid B (Sal-B), both of them having antifibrotic, antioxidative and anti-inflammatory properties [19,20]. Sal-A can inhibit cardiac and hepatic fibrosis by inhibiting fibroblast migration, blocking myofibroblast transformation, inhibiting collagen accumulation and decreasing the excessive activation of PI3K/Akt [21,22]. Sal-B attenuates cardiac and hepatic fibrosis by inhibiting fibroblast migration, collagen deposition and activation of MEK/ERK/p38 MAPK, NF-κB and TGF-β1/Smads pathways [20,23,24]. Sal B has also been reported to be effective against the malignant transformation of oral precancerous lesion [19]. In this study, we investigate the antifibrotic effect and mechanism of Tan-IIA1, Sal-A and Sal-B in mice oral mucosal fibroblasts (MOMFs) and human HaCaT cells *in vitro*. 

## 2. Results and Discussion

### 2.1. Tan-IIA1, Sal-A and Sal-B Ameliorated the ANE-Induced Abnormal Viability of MOMFs and Inhibited Collagen Accumulation

OSF is a fibrosing disorder of mouth, pharynx and esophagus with a malignant transformation rate of 2.3%–7.6% [25]. In our study, we have developed a chewable tablet to treat OSF, which mainly includes *Salvia miltiorrhiza*, *Panax notoginseng*, *Calculus bovis* and borneol [5,14]. In the previous study, we have reported the effect and mechanism of the major active compounds of *Panax notoginseng*, *Calculus bovis* and borneol on OSF [5,14,26]. In this study, we investigate the antifibrotic effect of Tan-IIA1, Sal-A and Sal-B, the important active compounds of *Salvia miltiorrhiza*.

**Figure 1:**
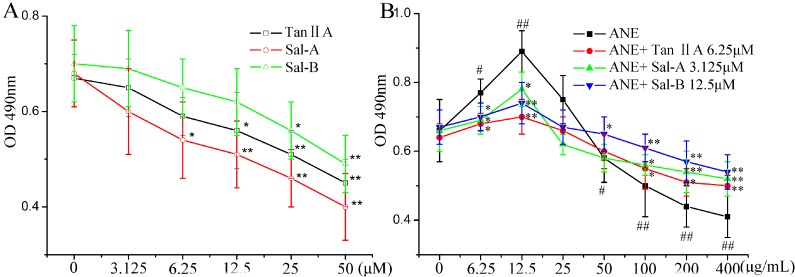
Tanshinone IIA1 (Tan-IIA1), salvianolic acid A (Sal-A) and Sal-B ameliorated the areca nut extract (ANE)-induced abnormal viability of mice oral mucosal fibroblasts (MOMFs). (**A**) The cytotoxicity of Tan-IIA1, Sal-A and Sal-B on MOMFs. After being exposed to Tan-IIA1, Sal-A and Sal-B for 48 h, the cell viability was measured by the MTT method; *n* = 5, * *p* < 0.05 and ** *p* < 0.01 *vs.* the 0 μM group. (**B**) The influence of Tan-IIA1, Sal-A and Sal-B on the ANE-induced abnormal viability of MOMFs. After being exposed to ANE (12.5 μg/mL) with or without Tan-IIA1 (6.25 μM), Sal-A (3.125 μM) and Sal-B (12.5 μM) for 48 h, the cell viability was measured by the MTT method. Data are expressed as the means ± SD. *n* = 5. ^#^ *p* < 0.05 and ^##^ *p* < 0.01 *vs.* the 0 μg/mL ANE group; * *p* < 0.05 and ** *p* < 0.01 *vs.* the group only treated with ANE.

As shown in Figure 1A, Tan-IIA1 at 6.25 μM, Sal-A at 3.125 μM and Sal-B at 12.5 μM showed no significant cytotoxicity after treatment for 48 h, and we took them as the test concentration for the following experiments. ANE at low concentrations (6.25–12.5 μg/mL) could significantly increase the viability of MOMFs, but at high concentrations (50–400 μg/mL), significantly decrease the viability of MOMFs. We took 12.5 μg/mL as the test concentration of ANE for the following experiments. Tan-IIA1, Sal-A and Sal-B could significantly revise the effect of ANE on the viability of MOMFs (Figure 1B).

**Figure 2:**
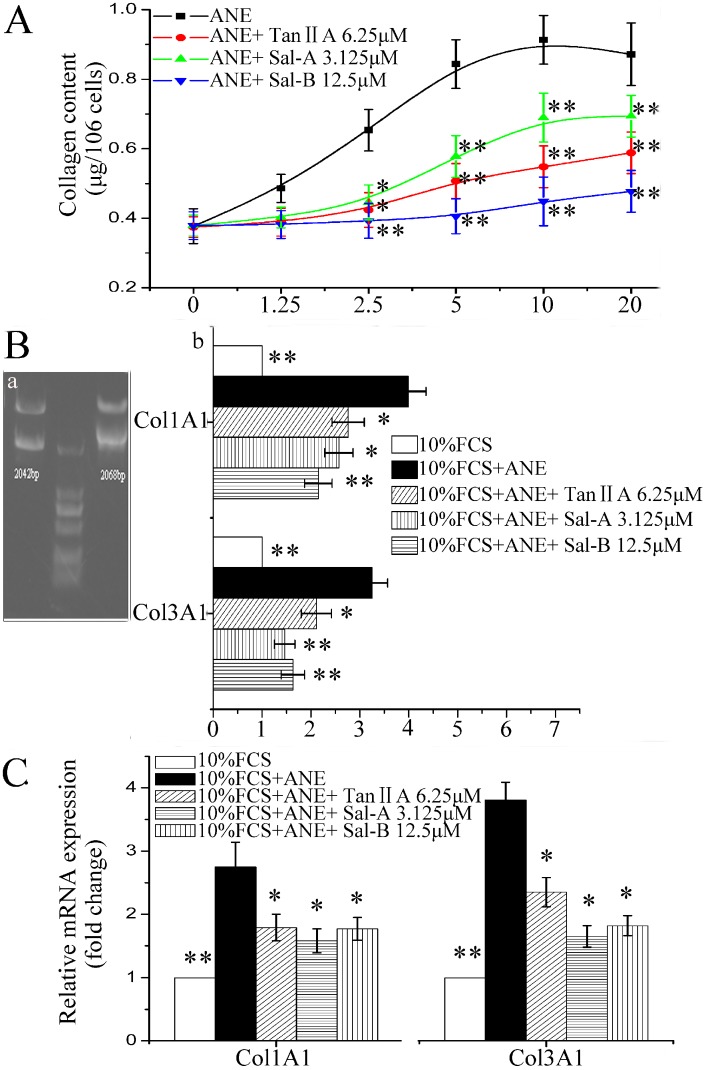
Tan-IIA1, Sal-A and Sal-B inhibited the ANE-induced collagen accumulation and transcription of procollagen genes *COL1A1* and *COL3A1*. (**A**) Tan-IIA1, Sal-A and Sal-B inhibited the ANE-induced collagen accumulation; *n* = 5, * *p* < 0.05 and ** *p* < 0.01 *vs.* the group only treated with ANE. (**B**) Tan-IIA1, Sal-A and Sal-B decreased the promoter activity of human procollagen genes *COL1A1* and *COL3A1.* (a) Double-enzyme digestion of pCol1A1-p-Luc and pCol3A1-p-Luc plasmids; (b) luciferase reporter plasmid assay; the results are shown as the fold change of the 10% FCS control. *n* = 5, * *p* < 0.05 and ** *p* < 0.01 *vs.* the control containing 10% FCS and ANE (12.5 μg/mL). (**C**) qRT-PCR assay. MOMFs were treated as in the gelatin zymography assay. The 2^−ΔΔCt^ values were calculated, and the results are expressed as the fold change of the 10% FCS control. *n* = 5, * *p* < 0.05 and ** *p*< 0.01 *vs.* the control containing 10% FCS and ANE (12.5 μg/mL).

ANE has been reported to induce the accumulation of ECM [7]. In our study, ANE (2.5–20 μg/mL) could significantly induce collagen accumulation, and Tan-IIA1, Sal-A and Sal-B could significantly decrease the ANE-induced collagen accumulation (Figure 2A). In addition, Tan-IIA1, Sal-A and Sal-B could significantly decrease the promoter activity of human procollagen genes *COL1A1* and *COL3A1* determined by luciferase reporter plasmid assay (Figure 2B) and decrease the transcription of *COL1A1* and *COL3A1* in MOMFs determined by qRT-PCR assay (Figure 2C). The primers for qRT-PCR assay are listed in Table 1.

**Table 1:** The sequences of the primers used for qRT–PCR analysis.

Gene	Forward	Reverse
COL1A1	5'-GTGCTAAAGGTGCCAATGGT-3'	5'-ACCAGGTTCACCGCTGTTAC-3'
COL3A1	5'-AGGGGAGCTGGCTACTTCT-3'	5'-CCTCCTTCAACAGCTTCCTG-3'
MMP-1	5'-CTTCTTCTTGTTGAGCTGGACTC-3'	5'-CTGTGGAGGTCACTGTAGACT-3'
MMP-13	5'-CTTCTTCTTGTTGAGCTGGACTC-3'	5'-CTGTGGAGGTCACTGTAGACT-3'
TIMP1	5'-TGACATCCGGTTCGTCTACA-3'	5'-TGCAGTTTTCCAGCAATGAG-3'
TIMP2	5'-AGTTCTTCGCCTGCATCAAG-3'	5'-GTCGAGAAACTCCTGCTTGG-3'
CTGF	5'-TCAACCTCAGACACTGGTTTCG-3'	5'-TAGAGCAGGTCTGTCTGCAAGC-3'
TGF-β1	5'-AAACGGAAGCGCATCGAA-3'	5'-GGGACTGGCGAGCCTTAGTT-3'
IL-6	5'-TTCCATCCAGTTGCCTTCTT-3'	5'-ATTTCCACGATTTCCCAGAG-3'
IL-1	5'-CCCAAGCAATACCCAAAGAA-3'	5'-CATCAGAGGCAAGGAGGAAA-3'
TNF-a	5'-CAAATGGCCTCCCTCTCAT-3'	5'-TGGGCTACAGGCTTGTCACT-3'
β-actin	5'-GGAGACAACCTGGTCCTCAG-3'	5'-ACCCAGAAGACTGTGGATGG-3'

### 2.2. Tan-IIA1, Sal-A and Sal-B Improved MMP-2/-9 Activity and Inhibited TIMP-1/-2 Expression after ANE Treatment

**Figure 3:**
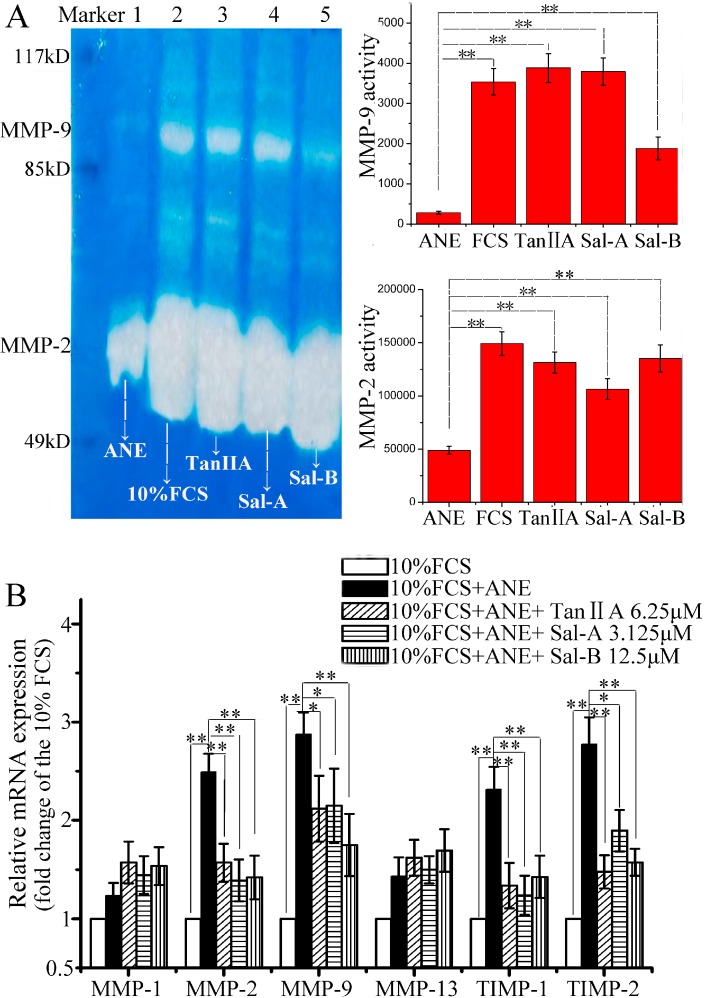
Effect of Tan-IIA1, Sal-A and Sal-B on MMP activity and TIMP expression after ANE treatment. (**A**) Effect of Tan-IIA1, Sal-A and Sal-B on MMP-2/-9 activity (left: the graph of the gelatin zymography assay; right: the statistical results). MOMFs were cultured in 10% FCS DMEM medium with or without ANE (12.5 μg/mL) and simultaneously treated with or without Tan-IIA1 (6.25 μM), Sal-A (3.125 μM) and Sal-B (12.5 μM). Lane 1 (ANE-only treated control): FCS + ANE; Lane 2 (blank control): FCS; Lane 3 (Tan-IIA1-treated group): FCS + ANE + Tan-IIA1; Lane 4 (Sal-A-treated group): FCS + ANE + Sal-A; Lane 5 (Sal-B-treated group): FCS + ANE + Sal-B. *n* = 3, ** *p* < 0.01 *vs.* the ANE only-treated control. (**B**) Effect of Tan-IIA1, Sal-A and Sal-B on the expression of MMP-1, MMP-2, MMP-9, MMP-13, TIMP-1 and TIMP-2 by qRT-PCR assay; the result was shown as the fold change of the 10% FCS group. *n* = 5, * *p* < 0.05 and ** *p* < 0.01 *vs.* the FCS + ANE group.

The homeostatic equilibrium between synthesis and degradation of ECM is directly regulated by MMPs and TIMPs. However, till now, the research results about the effect of ANE or arecoline, the major active compound of ANE, on the activity of MMPs and the expression of TIMPs were controversial. Chang Y. C. *et al.* have reported that the cells cultured from OSF specimens are found to have higher TIMP-1 expression than normal buccal mucosal fibroblasts and that arecoline can elevate TIMP-1 expression and inhibit MMP-2 activity in a dose-dependent manner *in vitro*. They think that the increased TIMP-1 expression and inhibition of MMP-2 activity in buccal mucosal fibroblasts is the possible mechanism for OSF [27]. Shieh D. H. *et al.* have found that arecoline acts not only as an inhibitor on the gelatinolytic activity of MMP-2, but also a stimulator for TIMP-1 activity. They think that the decrease of collagen phagocytosis and the augmented expression of TIMP-1 induced by arecoline in human buccal mucosal fibroblasts is a possible pathogenesis for OSF [28,29]. Xia L. *et al.* have shown that arecoline at 20 μg/mL decreases the activity of MMP-2 in human oral primary fibroblasts *in vitro* [30]. In our study, we have also found that ANE at 12.5 μg/mL could decrease MMP-2/-9 activity and increase TIMP-1/-2 expression, but almost no effect on the expression of MMP-1 and MMP-13; Tan-IIA1, Sal-A and Sal-B could revise the effect of ANE on MMP-2/-9 activity and TIMP-1/-2 expression (Figure 3).

However, there are also some reports showing that ANE elevates MMPs activity and decreases TIMPs production. Chang M. C. *et al.* have shown that ANE at 800 and 1200 μg/mL increases MMP-9 activity and suppresses TIMP-1/-2 production, but arecoline at 0.2 and 0.8 mmol/L decreases MMP-9 activity, in SAS epithelial cells [31]. In their study, the effects of ANE and arecoline on MMP-9 activity are paradoxical; in addition, the concentrations of ANE and arecoline that they used are too high, because when exceeding 50 μg/mL, ANE will show significant cytotoxicity (Figure 1); this result is similar to another report [8]. Tseng, Y. H. *et al.* have shown that areca nut components can activate MMP-9, but not MMP-2, in cultured gingival keratinocytes [32]. As for these controversial results, the reasons may be complex. First, areca nuts contain polyphenols, arecoline, arecaidine and other alkaloids; the content of these compounds in the samples obtained from different places may be different. Secondly, the cells the research groups used are different and may influence the results. Finally, the concentrations of ANE that the research groups used are also different.

### 2.3. Tan-IIA1, Sal-A and Sal-B Inhibited the Activation of AKT and ERK MAPK Pathways and Influenced the Release of Cytokines in MOMFs after ANE Treatment

**Figure 4:**
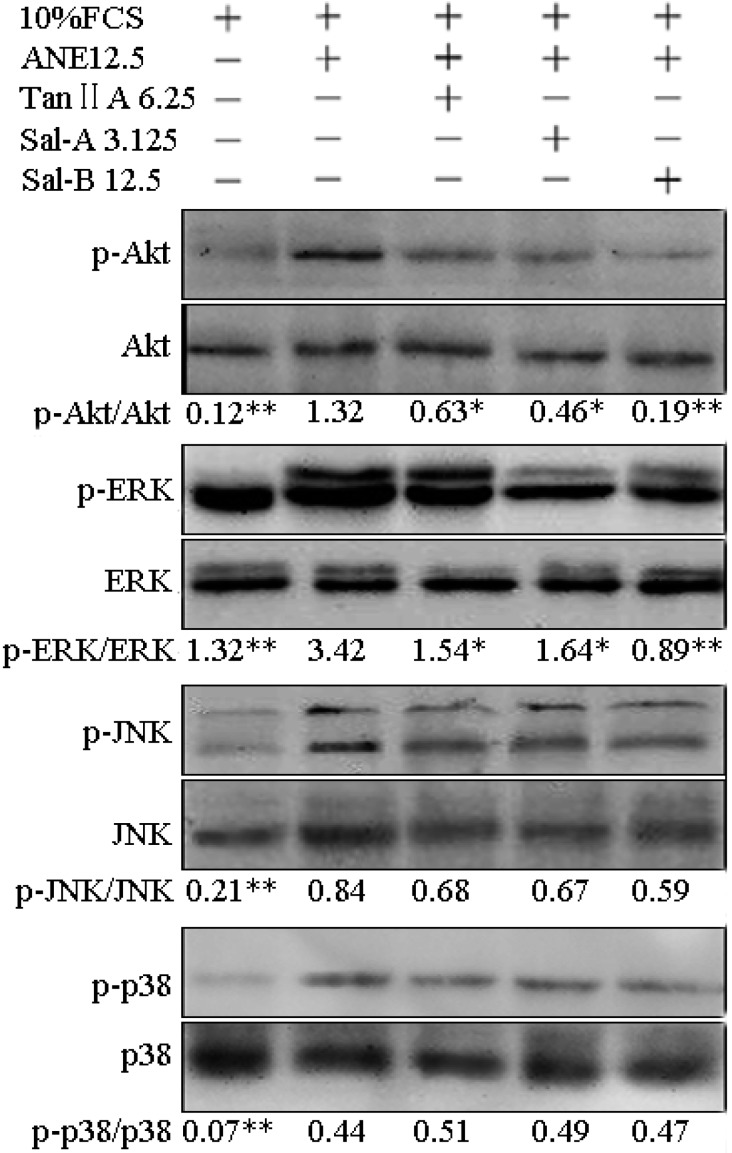
Effect of Tan-IIA1, Sal-A and Sal-B on the ANE-induced activation of AKT and ERK/JNK/p38 MAPK pathways. MOMFs were exposed to ANE (12.5 μg/mL), with or without Tan-IIA1 (6.25 μM), Sal-A (3.125 μM) and Sal-B (12.5 μM) for 48 h. *n* = 3, * *p* < 0.05 and ** *p* < 0.01 *vs.* the FCS + ANE group.

**Figure 5:**
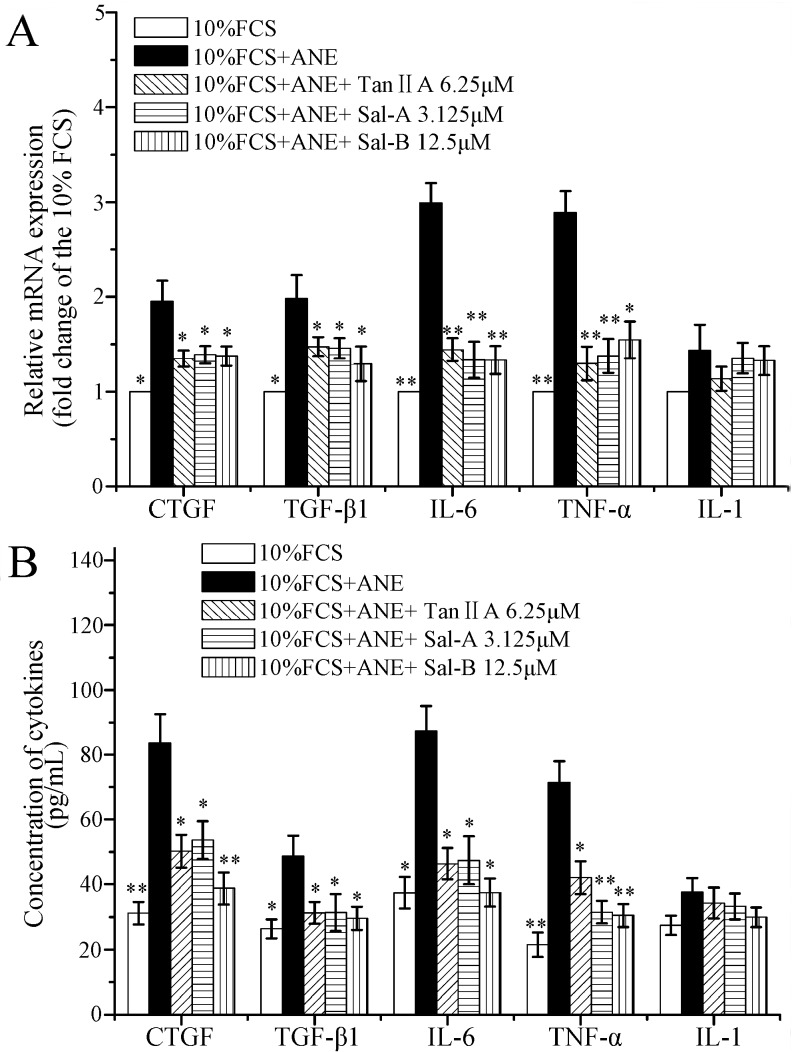
Influence of Tan-IIA1, Sal-A and Sal-B on the transcription and release of cytokines after ANE treatment. MOMFs were exposed to ANE (12.5 μg/mL), with or without Tan-IIA1 (6.25 μM), Sal-A (3.125 μM) and Sal-B (12.5 μM) for 48 h. The cells were harvested and used in qRT-PCR assay (**A**); and the supernatants were collected and used in ELISA assay (**B**). *n* = 5, * *p* < 0.05 and ** *p* < 0.01 *vs.* the FCS + ANE group.

It has been reported that ANE can activate PI3K/Akt, ERK/JNK/p38 MAPK and NF-κB pathways [9,11,12,13], and the activation of these signaling pathways plays important roles in OSF and oral carcinoma [8,9]. In the previous reports, Tan-IIA1, Sal-A and Sal-B have been shown to be able to ameliorate hepatic, pulmonary and cardiac fibrosis by suppressing the activation of PI3K/AKT, p38/ERK/JNK, NF-κB and TGF-β/Smad pathways [17,18,20,22,23,24]. In our study, ANE was also shown to significantly elevate the phosphorylation levels of p-Akt, p-ERK, p-JNK and p-p38, whereas Tan-IIA1, Sal-A and Sal-B significantly decreased the ANE-induced activation of Akt and ERK pathways (Figure 4). In addition, OSF is a chronic inflammatory disease. CTGF, IL-6, IL-1 and TNF-α have been proven to play important roles in OSF [8], and ANE has been shown to be able to induce prostaglandin E2, IL-6, TGF-β and TNF-α production [33,34]. In our study, we found that Tan-IIA1, Sal-A and Sal-B could inhibit the transcription and release of CTGF, TGF-β, IL-6 and TNF-α in MOMFs after ANE treatment (Figure 5).

### 2.4. Tan-IIA1, Sal-A and Sal-B Inhibited the Release of TGF-β and Activation of TGF-β/Smads Pathway in HaCaT Cells

**Figure 6:**
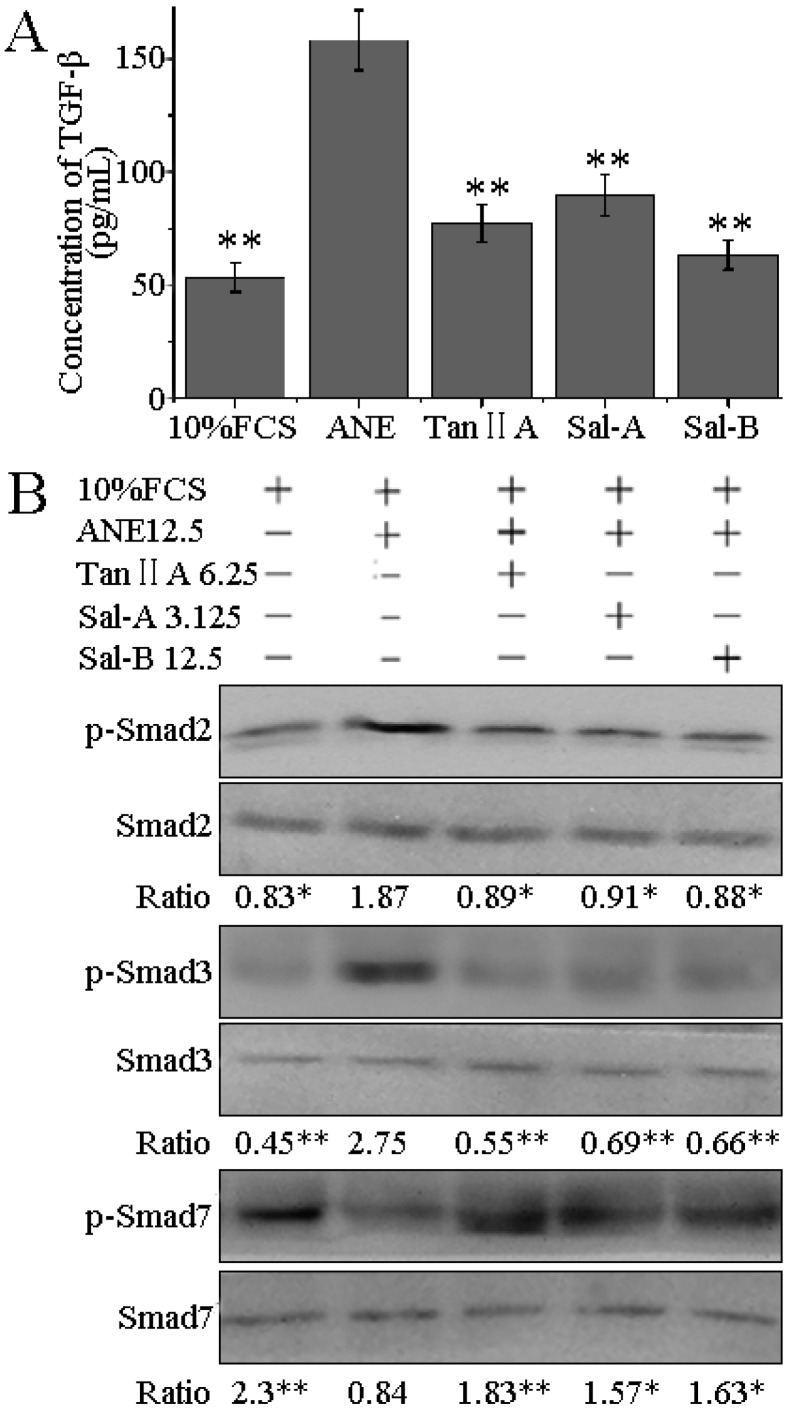
Effect of Tan-IIA1, Sal-A and Sal-B on the ANE-induced release of TGF-β and activation of TGF-β/Smads pathway. HaCaT cells were exposed to ANE (12.5 μg/mL), with or without Tan-IIA1 (6.25 μM), Sal-A (3.125 μM) and Sal-B (12.5 μM) for 48 h. The supernatants and cells were collected and used in ELISA (**A**) and Western blot assays (**B**), respectively. *n* = 3, * *p* < 0.05 and ** *p* < 0.01 *vs.* the FCS + ANE group.

Although fibroblast is an important cell type, which is responsible for collagen metabolism, in view of the anatomy, the oral epithelium is the first line of defense against ANE stimuli. During chewing of betel quid, epithelium is first exposed to areca nut components, and later, the areca nut components get diffused into the submucosal region; oral keratinocytes might affect collagen metabolism of fibroblasts by an indirect way. Xia L. *et al.* have shown that when cocultured with keratinocytes pretreated by arecoline, fibroblasts produce more soluble collagen and TIMP-1 [30]. Khan I. *et al.* have shown that oral epithelium plays important roles in OSF, and ANE can induce the release of TGF-β and activate Smad signaling pathway in human keratinocyte HaCaT cells. They think that areca nut components and TGF-β can diffuse into the submucosal region and synergize to activate fibroblasts [8]. In our study, we also determined the effect of Tan-IIA1, Sal-A and Sal-B on TGF-β/Smads signaling pathway in HaCaT cells, and found that ANE could significantly increase the release of TGF-β, elevate the levels of p-Smad2 and p-Smad3 and decrease the level of p-Smad7, whereas Tan-IIA1, Sal-A and Sal-B could significantly inhibit the release of TGF-β and activation of Smads signaling pathway in HaCaT cells (Figure 6).

## 3. Experimental Section

### 3.1. Material

Tanshinone IIA1 (Tan-IIA1, C_19_H_18_O_3_), salvianolic acid A (Sal-A, C_26_H_22_O_10_) and salvianolic acid B (Sal-B, C_36_H_30_O_16_) were purchased from Sigma-Aldrich (Milwaukee, WI, USA). Areca nut (*Areca catechu* L.) was purchased from Haozhou medicine market (Haozhou, China). The specimen was identified by Bang-Xing Han (Jiangsu University, Zhenjiang, China) and deposited in our lab. The areca nut extract (ANE) was prepared according to the “Pharmacopoeia of the People’s Republic of China 2005”; the content of arecoline was 0.31%, determined by HPLC assay (LC200 HPLC, JASCO, Tokyo, Japanese). The HPLC assay was accomplished with a C18 column (Spherisorb ODS2, 250 × 4.6 mm, 5 µm, Brookline, MA, USA) fitted with a UV-visible detector (UV-1575) at 216 nm, using a 10 mM ammonium acetate (pH 4.5)/acetonitrile (90:10, v/v) solution as a mobile phase at a flow rate of 1.0 mL/min at 35 °C. A volume of 10 μL was injected into the column. The limit of quantification (S/N = 10) was 1.23 µg/mL; the limit of detection (S/N = 3) was 0.41 µg/mL; the average recovery was 97.66%; and the retention time was 8.16 min.

### 3.2. Cell Culture and MTT Assay

Male and female Balb/c mice (20 ± 2 g) were kept in a 12-h light/dark cycle at 22 ± 2 °C with water and food *ad libitum*. The experiments were conducted in accordance with the Guide for the Care and Use of Laboratory Animals, Shantou University Medical College. Mice were killed by cervical dislocation, and oral mucosa was stripped under sterile conditions. Mice oral mucosal fibroblasts (MOMFs) were cultured as the previous report [5]. Human keratinocyte HaCaT cells were cultured in 10% FCS DMEM medium. The influence of Tan-IIA1, Sal-A, Sal-B and ANE on the viability of MOMFs and HaCaT cells was measured by the MTT method [5]. The serial dilutions of Tan-IIA1, Sal-A, Sal-B (0, 3.125, 6.25, 12.5, 25, 50 μM) and ANE (6.25, 12.5, 25, 50, 100, 200, 400 μg/mL) were made in 10% FCS DMEM medium (DMSO ≤ 0.5%).

### 3.3. Plasmid Construction, Transfection and Luciferase Assay

To construct human procollagen gene promoter luciferase reporter plasmids, the promoters of human procollagen genes *COL1A1* (NW_004078092.1, −2000 to + 42 bp) and *COL3A1* (NW_004078008.1, −2031 to +37 bp) were cloned into pGL3-basic plasmid and named the pCol1A1-p-Luc and pCol3A1-p-Luc plasmid, respectively. The primers to colon *COL1A1* promoter were F: 5'-AAAGGTACCACATATGGGGAGGGGCGGGGAGC-3', R: 5'-AGGCTCGAGCCTCTTGGC CGTGCGTCAGGA-3'. The primers to colon *COL3A1* promoter were F: 5'-GGGGGTACCTGCATA TAACAGCCTTTCCCCAG-3', 5'-AAACTCGAGAGTGGGATGAAGCAGAGCGAG-3'. Human HaCaT cells (1 × 10^4^/well) were seeded in 96-well microplates and transfected with pCol1A1-p-Luc or pCol3A1-p-Luc plasmids using the Lipofectamine 2000 reagent (Invitrogen, Carlsbad, CA, USA). A pRL-CMV plasmid was used as the internal control. After a 6-h incubation at 37 °C, the cells were treated with or without ANE (12.5 μg/mL) and simultaneously treated with or without Tan-IIA1 (6.25 μM), Sal-A (3.125 μM) and Sal-B (12.5 μM). After 24 h, the luciferase activity was determined using the Luciferase Reporter Assay Kit (BD Biosciences Clontech, Franklin Lakes, NJ, USA).

### 3.4. Hydroxyproline Assay

MOMFs (1 × 10^5^/well) were seeded in 24-well plates for 24 h, then exposed to 10% FCS DMEM containing ANE (1.25, 2.5, 5, 10, 20 μg/mL), with or without Tan-IIA1 (6.25 μM), Sal-A (3.125 μM) and Sal-B (12.5 μM) for 48 h. The supernatants were collected, and the hydroxyproline content was determined by the chloramine T method [5]. Data were expressed as micrograms of collagen of 10^6^ cells, assuming that collagen contains 13.5% hydroxyproline.

### 3.5. Gelatin Zymography Assay

MOMFs (1 × 10^6^/well) in 6-well plates were exposed to 10% FCS DMEM containing ANE (12.5 μg/mL), with or without Tan-IIA1 (6.25 μM), Sal-A (3.125 μM) and Sal-B (12.5 μM). After 48 h, the supernatants were harvested, centrifuged for 15 min at 12,000× *g*, stored at −70 °C and used in gelatin zymography and ELISA assays. The cells were harvested and used in qRT-PCR and Western blot assays. The bands of MMP-2/-9 were quantified by scanning densitometry, and the activity was expressed as the multiplication product of the zone area and average gray value [5].

### 3.6. qRT-PCR Assay

The cells were treated as in the gelatin zymography assay. The total RNA was extracted using the TRIzol^®^ Plus RNA purification kit (Invitrogen). DNA contamination was removed with the addition of DNase I (Invitrogen). Total RNA was eluted in nuclease-free water and quantified spectrophotometrically at 260 nm. First-strand cDNA was synthesized by reverse transcription using oligo(dT)18 primer (Invitrogen). The primers for qRT-PCR assay are listed in Table 1. The values of 2^−ΔΔCt^ of each group were calculated, and the results were expressed as the fold change of the control.

### 3.7. ELISA Assay

The cells were treated as in the gelatin zymography assay; the levels of CTGF, TGF-β1, IL-6, TNF-α and IL-1 were quantitatively measured by specific ELISA kits performed according to the manufacturer’s instructions (Beijing Dakewe Biotech Lit. Company, Beijing, China).

### 3.8. Western Blot Assay

Antibodies against Akt, p-Akt (Thr308), ERK, p-ERK, JNK, p-JNK, p38, p-p38, Smad2, p-Smad2, Smad3, p-Smad3, Smad7, p-Smad7 and peroxidase-conjugated antibodies (anti-rabbit or anti-goat) were purchased from Cell Signaling Technology, Inc. (Danvers, MA, USA). The cells were treated as in the gelatin zymography assay. Twenty milligrams of whole protein extracts were subjected to SDS-PAGE. The results were quantified by scanning densitometry, as previously reported [35].

### 3.9. Statistical Analysis

Statistical differences between experimental groups were determined by one-way analysis of variance (ANOVA) using SPSS 13.0 software. Data are the mean ± standard deviation (mean ± SD).

## 4. Conclusions

In conclusion, Tan-IIA1, Sal-A and Sal-B can inhibit the ANE-induced abnormal viability and collagen accumulation of MOMFs, inhibit the transcription of procollagen genes, increase MMP-2/-9 activity, decrease TIMP-1/-2 expression and suppress the ANE-induced high expression of cytokines. These activities of these three compounds may be based on their mechanisms, by which they can inhibit the ANE-induced activation of AKT, ERK MAPK and TGF-β/Smads pathways. Finally, our research has shown that Tan-IIA1, Sal-A and Sal-B possess excellent antifibrotic activity *in vitro*, and we think that it is possible to use these compounds to promote the rehabilitation of OSF patients. 
